# Teaching Social Hygiene

**Published:** 1913-10

**Authors:** 


### Teaching Social Hygiene.

The Buffalo Congress on School Hygiene has taken a definite stand for instruction in the facts of sex. The fallacious old theory that ignorance means innocence has been abandoned as far as this great meeting of distinguished educationalists is concerned. The prevalent cowardice in the face of vice and its consequences has endangered the welfare of the human race, in their opinion, and nothing less than a revolt from the common methods of shunning the truth will suffice to meet the situation.

Ignorance, even if it existed, would not mean innocence. It would simply mean defenselessness. To turn young persons of either sex out into the hurly-burly of life unfortifiefid by knowledge of their physical natures and the perils which passion creates is as senseless as to drive a spring lamb among a flock of hungry wolves, and the consequences are commonly much the same. But in most cases the ignorance which foolish parents and teachers foster so assiduously is purely imaginary. It does not exist. It is only in the most exceptional cases that young people fail to learn the facts of sex in their tender years, but instead of coming, as it ought, from their wise elders, it comes far too often from depraved associates. Knowledge flows from a poisoned spring. The immaculate ignorance of which we hear so much praise from those who would hide the light is gross humbug and most of the pleas for it are little better than cant.

But instruction in physiology and hygiene, essential as they are, will not alone suffice to cure the evils that arise. No matter how much the individual may know he is still in peril unless his will is educated. Side by side with the instruction in those truths which are vital to wholesome living should proceed training of the will. As the Jesuit Father Tiernay expressed it to the congress, "Teach that purity is noble and possible, that vice is vile and carries its own punishment, that marriage is inviolable and that the family is sacred."

But, after all, such phrases as these ring a little hollow. These things may be taught and taught from now until Gabriel sounds his trumpet, and nothing will come of it unless we can master the magic art of transforming precept into volition.

Who will tell the world how to make young people act upon the good advice they receive? Who will tear away the deceptive veil of beauty that covers the hideousness of vice and inspire youth to resist its allurement? No doubt the task is difficult, but we can see one thing that might help. It is a radical reform of the theatres. Out of every ten plays that our young people flock to see, nine make vice attractive. The infidelity of husbands, the humor of drunkenness, the gaiety of brothels, the fascination of lewd intrigue, such are the materials from which many stage plays are constructed, and they are seldom depicted in their true squalor and misery, but almost always with a false witchery that plays into the hands of the tempter. The newer drama which represents vice in its true ugliness is often denounced as immoral, but it is an old trick of the devil to destroy his foes by defaming them when he can.

But if we could outwardly reform the stage and literature and conventional society, we should not even then have gone to the root of the matter. The real inspiration to resist vicious temptation and live a pure life is an inner fire. When it is lighted in the soul, knowledge acquires meaning and precepts reach down into the will. While it burns conduct marches on the high levels of life. When it dies men sink into the sloughs of wickedness.

Who shall light this inner fire in the souls of American youth? The Buffalo Congress might have desired a regeneration as well as a revolution. Where shall we look for the vital spark? Dr. Charles W. Eliot specifies three "principal causes of the present evil conditions—lust in men, lack of moral principle in some women, and the depravity of those who trade in lust and immorality." Nobody can say a word for commercialized vice. It should be extirpated from the world. All will agree with Dr. Eliot that it

"should be attacked with all the forms and all the powers of the law." The only hope lies in annihilating the business.

But when we begin to consider the elimination of lust, we stand on ground not quite so safe. In our zeal for reform we must be careful not to run counter to the laws of the universe. Biology laughs at the notion of producing a passionless male, or one in whom the sexual impulse has been chilled. With all our striving we cannot stay the action of the law of survival, and nature will inevitably select the men for parents in whom passion is the strongest. This fact is fundamental.

To weaken the paternal impulse, which we may stigmatize as lust if we wish, is to retire unconquered from the struggle for existence and leave the world to be inherited by those who obey nature's law. Safety lies not in eliminating passion, but in controlling it. Readers of "The Wandering Jew" will remember a remarkable passage in which the general of the Jesuits describes the effects of chastity. He had not by any means eliminated his natural passions, but he had reined and harnessed them, and by virtue of their transformed potency he was master of the civilized world.

The Biblical writers knew all about this. "He that conquers himself is greater than he that taketh a city," said one of them, anticipating Eugene Sue's rule for achieving power. So the teacher of sexual hygiene has to sail perilously between Scylla and Charybdis. If he decries passion too much, he saps the springs of race vigor. If he fails to slay vice in its wolfishness he sees the old tragedy repeated of Saturn devouring his children.

What is he to do? One is almost driven to remember an ancient saying to which William James gave new meaning for the modern world, "Ye must be born again." Wriggle and twist as we will, are we not finally compelled to turn to religion for refuge? But what is religion? That is often the rub.—*The Portland (Oregon) Oregonian.*

---

Big Game.—"A penny mouse trap, please. And let me have it quickly, as I want to catch a train."—*London Opinion.*

---

Little Muriel flew into the house, flushed and breathless.

"Oh, mother!" she cried, "don't scold me for being late for tea, for I've had such a disappointment. A horse fell down and they said they were going to send for a horse doctor, so, of course, I waited, he came, and oh! mother, what do you think? It wasn't a horse doctor at all. It was only a man."—*Tit-Bits.*

